# Intravesical Resiniferatoxin for the Treatment of Storage Lower Urinary Tract Symptoms in Patients with Either Interstitial Cystitis or Detrusor Overactivity: A Meta-Analysis

**DOI:** 10.1371/journal.pone.0082591

**Published:** 2013-12-20

**Authors:** Changcheng Guo, Bin Yang, Wenyu Gu, Bo Peng, Shengqiang Xia, Fengqiang Yang, Deyi Wen, Jiang Geng, Yuanyuan Zhang, Junhua Zheng

**Affiliations:** 1 Department of Urology, Shanghai Tenth People's Hospital, Tongji University School of Medicine, Shanghai, People's Republic of China; 2 Wake Forest Institute for Regenerative Medicine, Wake Forest University School of Medicine, Winston-Salem, North Carolina, United States of America; Oklahoma University Health Sciences Center, United States of America

## Abstract

**Background:**

While Resin­iferatoxin (RTX) has been widely used for patients with storage lower urinary tract symptoms (LUTS), its clinical efficiency hasn't yet been well evaluated. A meta-analysis was performed to evaluate the exact roles of intravesical RTX for the treatment of storage LUTS in patients with either interstitial cystitis (IC) or detrusor overactivity (DO).

**Methods:**

A meta-analysis of RTX treatment was performed through a comprehensive search of the literature. In total, 2,332 records were initially recruited, 1,907 from Elsevier, 207 from Medline and 218 from the Web of Science. No records were retrieved from the Embase or Cochrane Library. Seven trials with 355 patients were included and one trial was excluded because of the lack of extractable data. The analyses were all performed using RevMan 5.1 and MIX 2.0.

**Results:**

Bladder pain was significantly reduced after RTX therapy in patients with either IC or DO. The average decrease of the visual an alogue pain scale was 0.42 after RTX treatment (p = 0.02). The maximum cystometric capacity (MCC) was significantly increased in patients with DO (MCC increase, 53.36 ml, p = 0.006) but not in those with IC (MCC increase, −19.1 ml, p = 0.35). No significant improvement in urinary frequency, nocturia, incontinence or the first involuntary detrusor contraction (FDC) was noted after RTX therapy (p = 0.06, p = 0.52, p = 0.19 and p = 0.41, respectively).

**Conclusions:**

RTX could significantly reduce bladder pain in patients with either IC or DO, and increase MCC in patients with DO; however, no significant improvement was observed in frequency, nocturia, incontinence or FDC. Given the limitations in the small patient size and risk of bias in the included trials, great caution should be taken when intravesical RTX is used before a large, multicenter, well-designed random control trial with a long-term follow-up is carried out to further assess the clinical efficacy of RTX in in patients with storage LUTS.

## Introduction

Storage lower urinary tract symptoms (LUTS) collectively represent a common condition, including bladder pain, increased urinary frequency, nocturia, urgency and incontinence, which have a major, deleterious, and bothersome impact on quality of life in patients with interstitial cystitis (IC) or detrusor overactivity (DO). The mechanism of storage LUTS in these patients is still not completely understood [1], and no standard treatment has been available for these patients. The current therapeutic approaches to control LUTS are clinically and scientifically unsatisfactory. The most commonly used oral drugs, such as pentosan polysulfate sodium, anti-inflammatory and anti-cholinergic agents, have limited efficiency and are usually associated with troublesome side effects [2]. It is still an unsolved enigma for physicians to treat LUTS patients effectively [3].

Recently, accumulated evidence suggests that dysfunction of afferent innervation of the bladder is involved in storage LUTS [4]. Unmyelinated sensory C-fibers constitute the majority of bladder afferents (about 70% in rat and 50% in human), and terminate in the detrusor muscle, submucosa and urothelium [5], [6]. Afferent C fibers are relatively inactive in adults during normal voiding, but can be abnormally activated by a variety of neurotransmitters and chemical mediators released from the detrusor and urothelium under IC or DO conditions, leading to bladder segmental contractility and subsequent development of storage LUTS [3], [7]–[9].

The transient receptor potential vanilloid type 1 (TRPV1), a nonspecific Ca^2+^ channel previously known as vanilloid receptor, is abundantly expressed in the bladder sensory C fibers and urothelial cells [10]. In patients with neurogenic DO, TRPV1 immunoreactive suburothelial nerve density is significantly increased [11], [12]. Increased TRPV1 can up-regulate the frequency of bladder reflex contractions, either via direct excitation of sensory C fibers or via urothelial-sensory fiber cross talk involving the release of neuromediators from the epithelial cells [13]. Consequently, a blockade of bladder C fiber sensory input might have the potential of inhibiting bladder reflex contractions, and improving storage LUTS in patients with IC or DO [9].

Resin iferatoxin (RTX) is a specific ligand of the TRPV1 receptor. Once activated by RTX, TRPV1 allows a massive Ca^2+^ and Na^+^ inflow into the neuron [14]. A high level of intracellular Ca^2+^ concentration is able to arrest voltage-sensitive Ca^2+^ conductance, disrupt the critical cellular metabolic pathways and release neuropeptides, such as calcitonin gene-related peptide (CGRP) and substance P (SP), which are accumulated in peripheral nerve endings. In the bladder, these acute effects are followed by a prolonged period during which the TRPV1 expression is remarkably decreased [15]. Following RTX binding to TRPV1, bladder afferent C fibers become inactivated through the desensitization process, after which bladder sensory input will be prevented from reaching the spinal cord [16]. Reports indicate that delivery of RTX could produce strong enduring analgesia in models with the spinal cord injury [17]. It was also reported that intravesical application of RTX could produce a significant decrease of TRPV1 immunoreactive suburothelial nerve fibers in patients with neurogenic DO [8], [11], [12]. For these reasons, intravesical desensitization of TRPV1 with RTX had been ever considered as a potential treatment for patients with storage LUTS [13], [18].

Several studies had been carried out to investigate the effect of intravesical RTX for the treatment of storage LUTS in IC and DO patients; however, most of these trials were retrospective analyses with case series, and only a few perspective studies with a very small sample size of participants in a single center were reported. Moreover, the results were highly inconsistent and prevented strong conclusions from being drawn [11], [12], [16], [18]–[32]. To further evaluate the potential of using RTX as a treatment for LUTS in patients with either IC or DO, we summarized and performed a meta-analysis of the current literature concerning the use of RTX for the treatment of storage LUTS in patients with either IC or DO. The evidence-based findings could provide urologists with useful information on the exact roles of intravesical RTX for the treatment of these patients.

## Materials and Methods

### 1. Search Strategy

**Figure 1:**
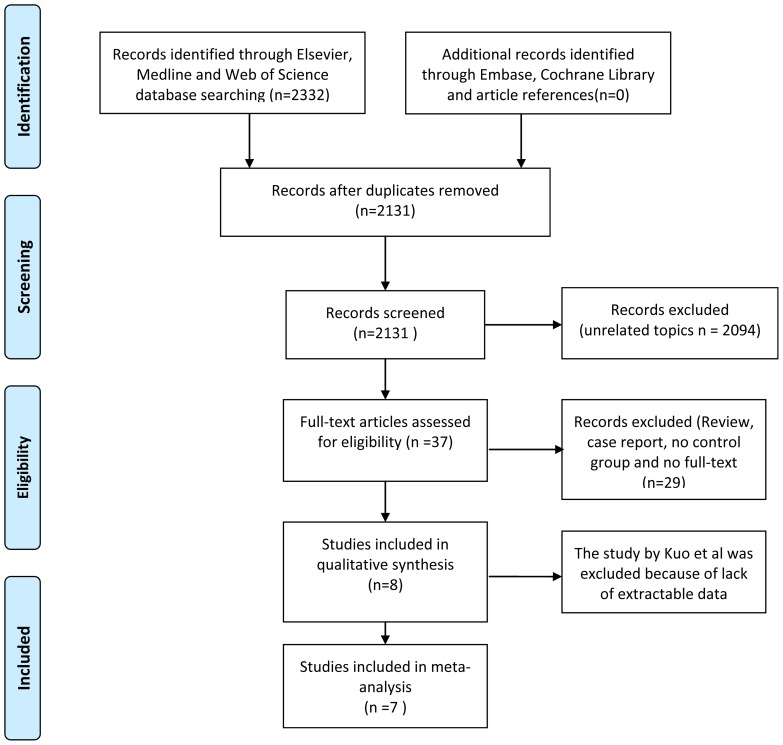
Flowchart of literature searches and results. In this meta-analysis, eight studies were selected for qualitative analysis. Of these eight studies, seven eligible controlled trials with 355 patients were included in our meta-analysis while the remaining one trial was excluded because of the lack of extractable data.

A literature search was performed in February 2013 using the Elsevier, Medline, Web of Science, Embase and Cochrane Library. The Elsevier and Medline searches included only a free-text protocol using the term Resiniferatoxin across the “Title’’ and ‘‘Abstract’’ fields of the records. Additionally, the following limits were used: humans and language (English). The searches of the Web of Science, Embase and Cochrane Library used the same free-text protocol and the same key words, applying no limits. All papers published since 2000 were taken into consideration. A total of 2,332 records were initially recruited in this study, with 1,907 records being retrieved from the Elsevier database, 207 records from the Medline database and 218 records from the Web of Science. No records were retrieved from the Embase or Cochrane Library. Three authors (Guo C, Yang B and Gu W) independently reviewed the records to select the studies comparing RTX and controls. Studies published only as abstracts and reports from academic meetings were not included in this analysis. Additionally, other relevant studies cited in the reference lists of the selected papers were evaluated. (Figure. 1).

### 2. Participants and Interventions

The inclusion criteria consisted of adult patients (male or female) with either IC or DO who had storage LUTS and were treated with either RTX or normal saline solution as the control. To be considered eligible, the study had to meet the following additional criteria: 1) All studies had to have a control group; 2) The baseline characteristics of patients from two arms had to be included; 3) The patients had to have a follow-up of at least four weeks; 4) The original data for dichotomous and continuous variables had to be provided or be calculable from the data source; 5) For studies with the same or overlapping data by the same authors, the more recent study with the greater number of subjects was chosen. With this criteria, eight studies were selected for qualitative analysis. Of these eight studies, seven eligible controlled trials with 355 patients were included in our meta-analysis while the remaining one trial was excluded because of the lack of extractable data (Figure. 1, Table 1).

**Table 1:** Characteristics of included trials.

First author year	kinds of LUTS	number of participants (R/N)	follow-up (month)	RTX (nM)	bladder pain (VAS score)		frequencey (times/day)		nocturia(times/night)		No. of incontinence (times/day)		MCC(ml)		FDC(ml)	
					B(R/N)	P(R/N)	B(R/N)	P(R/N)	B(R/N)	P(R/N)	B(R/N)	P(R/N)	B(R/N)	P(R/N)	B(R/N)	P(R/N)
Ham 2012	IC	18 (8/10)	3	_	3.3±0.8/3.5±1.1	2.9±0.4/3.3±0.7	16.8±6.7/15.6±7.6	15.4±2.9/14.8±6.0	4.4±1.7/4.4±2.5	3.6±1.5/3.1±1.4	_	_	210.4±34.7/239.0±36.0	222.9±36.9/242±50.2	_	_
Rios 2007	DO	53 (33/20)	1	50nM in 100ml	_	_	9.7±2.88/9.9±2.58	9.03±2.77/9.1±2.68	2.21±1.02/2.28±1.19	1.73±1.14/1.82±1.16	3.00± 2.46/5.06±3.37	2.68±3.08/4.5±3.83	287.5±181/240.6±85.8	298. ±161.5/270.5±109.6	103±60/124.3±73	120.2±68.9/140.8±84.4
Kuo 2006	DO	54 (26/28)	3	10nM in 30ml	_	_	_	_	_	_	*21.3± 11.7/20.1±13.0	*13.3±5.9/21.5±11.9	220±103/225±112	258±102/218±96	_	_
Payne 2005	IC	162 (119/43)	1	50nM in 50ml	6.4±1.3/5.8±1.6	4.57±1.38/4.7±1.6	_	_	_	_	_	_	_	_	_	_
Silva 2005	DO	28 (14/14)	3	50nM in 100ml	_	0.6±0.8/2±2.5	9.5±2.5/10±2	7.6±2.1/9.6±2.6	_	_	4.5 ±4.5/1.8 ±2.5	1.6±1.4/1±1.4	189±99/198±111	314±135/204±92	143±95/115±58	184±140/184±93
Chen 2005	IC	22 (18/4)	3	50-100nM in 50ml	_	3.86±3.1/5.68±1.7	_	17.47±14.38/16.9±9.64	_	3.4±3.14/5.75±7.21	_	_	_	_	_	_
Lazzeri 2000	IC	18 (9/9)	3	10nM in 30ml	5.55±0.29/5.77 ±0.28	4.77±1.99/5.66±0.71	12.44±0.71/11.66±0.71	10.44±3.36/12.55±2.13	3.77±0.28/3.88±0.22	3.11±0.71/3.66±1.18	_	_	_	_	_	_

R = patients treated with RTX, N =  controls, B =  the baseline symptom, P =  the post-treatment symptom.

“*” =  No. of incontinence episodes/3-day voiding diary, “−” =  data unavailable.

Data are expressed by mean ± standard deviation.

### 3. Data Extraction

Two investigators (Yang B and Gu W) independently extracted data, and all disagreements about eligibility were resolved by a third reviewer(Guo C) [33]. The following primary outcomes were evaluated in this review: bladder pain, urinary frequency, urgency, incontinence, nocturia, first involuntary detrusor contraction (FDC) and maximum cystometric capacity (MCC) (Table 1). Statistical analyses were performed by 2 independent authors (Yang F and Wen D) not involved in data collection. We extracted the records of post-treatment symptoms as the final data and expressed them by mean ± standard deviation (SD) to perform the meta-analysis. Estimated weighted mean differences were used for continuous variables. Inter-study heterogeneity was measured using the Q-test. Heterogeneity was also quantified with the I^2^ metric, which is independent of the number of studies included in the cumulative analysis. The I^2^ values range from 0% to 100%, with higher values denoting a greater degree of heterogeneity. Data was pooled using both fixed-effect and random-effect models. In the absence of inter-study heterogeneity, both the fixed-effect and random effect models can provide identical results, while in the presence of heterogeneity, the random-effect model was applied as it can give a more conservative estimate of the inter-study variance with a wider confidence interval (CI). The Begg's funnel plot was used to identify potential publication bias. In the Begg's funnel plot, an asymmetrical plot suggests a possible publication bias. Sensitivity analysis was carried out by study design, sample size, and the kind of disease. All the analyses were performed using the RevMan 5.1, and MIX 2.0 software packages. All *p* values were calculated using the 2-tailed student t test and *p* values < = 0.05 were considered statistically significant [34].

### 4. Quality Assessment

The quality of the included studies was assessed by 3 independent investigators (Peng B, Xia S and Geng J) according to the Cochrane Collaboration Reviewers' handbook and the Quality of Reporting of Meta-analysis Guidelines [35]. The quality items consisted of generation of randomization sequences, allocation concealment, description of withdrawals and dropouts, intent to treat analysis, incomplete outcome data, selective outcome reporting, evaluation of other possible biases and the blinding methods.

## Results

### 1. Outcomes of bladder pain

**Figure 2:**
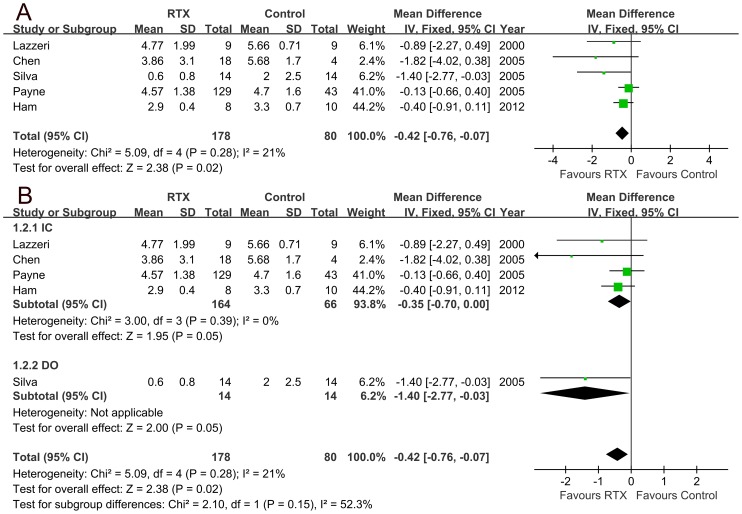
Visual an­alogue scale (VAS) score changes for bladder pain in all the patients (A; average decrease of VAS score 0.42, 95% CI 0.07 to 0.76, p = 0.02) and subgroup analyses in patients with either interstitial cystitis (IC) or detrusor overactivity (DO) (B; average decrease of VAS score for IC 0.35, 95% CI 0 to 0.7, p = 0.05; average decrease of VAS score for DO 1.4, 95% CI 0.03 to 2.77, p = 0.05).

Our data showed that bladder pain was significantly reduced after RTX therapy. The average decrease of the visual an­alogue pain scale (VAS) was 0.42 (95% CI 0.07 to 0.76, p = 0.02) after RTX treatment (Figure. 2A). Subgroup analyses indicated that RTX could significantly relieve bladder pain in patients with either IC or DO. The average decreases of VAS scores were 0.35 (95% CI 0 to 0.7, p = 0.05) and 1.4 (95% CI 0.03 to 2.77, p = 0.05) respectively after RTX treatment (Figure. 2B).

### 2. Outcomes of urinary frequency, nocturia and incontinence

**Figure 3:**
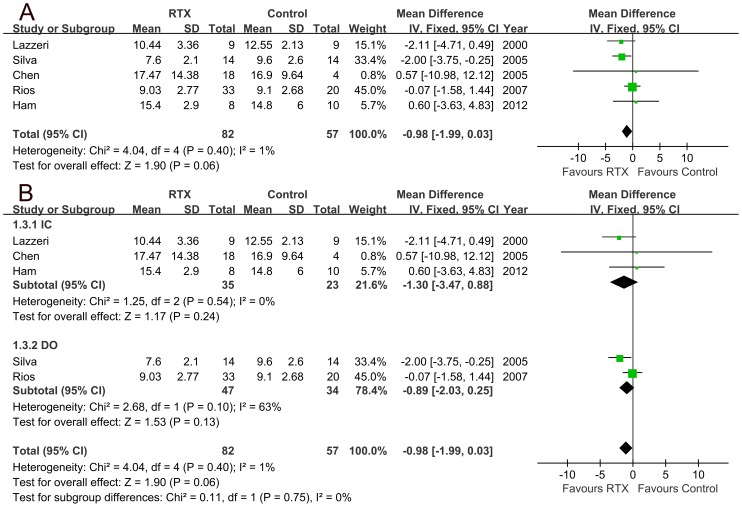
Urinary frequency changes in all the patients (A; mean difference 0.98, 95% CI −1.99 to 0.03, p = 0.06) and subgroup analyses in patients with either interstitial cystitis (IC) or detrusor overactivity (DO) (B; mean difference for IC −1.3, 95% CI −3.47 to 0.88, p = 0.24; mean difference for DO −0.89, 95% CI −2.03 to 0.25, p = 0.13).

**Figure 4:**
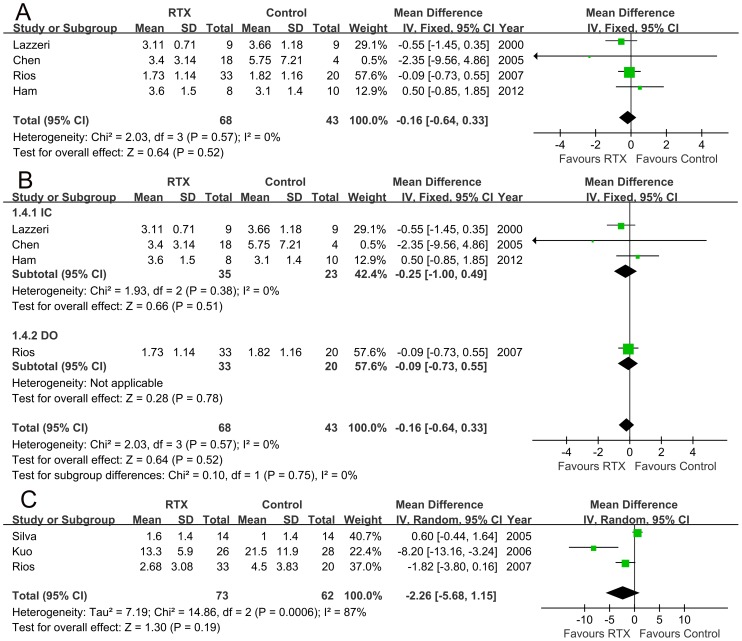
Nocturia changes in all the patients (A; mean difference −0.16, 95% CI −0.64 to 0.33, p = 0.52) and subgroup analyses of nocturia in patients with either interstitial cystitis (IC) or detrusor overactivity (DO) (B; mean difference for IC −0.25, 95% CI −1.00 to 0.49, p = 0.51; mean difference for DO −0.09, 95% CI −0.73 to 0.55, p = 0.78). Incontinence changes in all the patients (C; mean difference −2.26, 95% CI −5.68 to 1.15, p = 0.19).

Our data showed that no significant difference was found in urinary frequency between RTX treated and control groups (mean difference −0.98, 95% CI −1.99 to 0.03, p = 0.06, Figure. 3A). Subgroup analyses showed that RTX treatment could not significantly reduce urinary frequency in patients with IC (mean difference −1.3, 95% CI −3.47 to 0.88, p = 0.24, Figure. 3B), and no significant improvement was observed in the patients with DO (mean difference −0.89, 95% CI −2.03 to 0.25, p = 0.13, Figure. 3B). There was no significant difference in the decrease of nocturia between RTX treated and control groups (mean difference −0.16, 95% CI −0.64 to 0.33, p = 0.52, Figure. 4A). Subgroup analyses showed no significant improvement of nocturia for patients with IC (mean difference −0.25, 95% CI −1.00 to 0.49, p = 0.51, Figure. 4B) or patients with DO (mean difference −0.09, 95% CI −0.73 to 0.55, p = 0.78, Figure. 4B). No significant improvement was noted in urinary incontinence after RTX treatment (mean difference −2.26, 95% CI −5.68 to 1.15, p = 0.19, Figure. 4C).

### 3. Outcomes of urodynamic parameters

**Figure 5:**
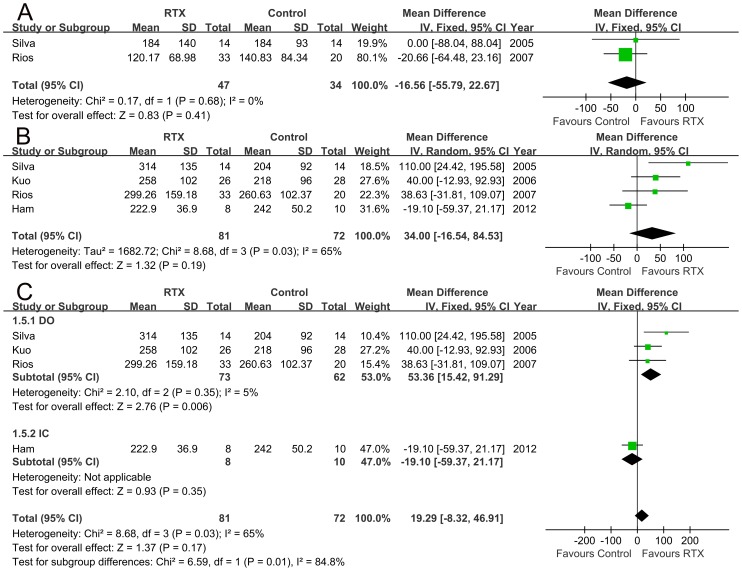
FDC (A; mean difference −16.56 ml, 95% CI −55.79 to 22.67 ml, p = 0.41) and MCC (B; mean difference 34ml, 95% CI -16.54 to 84.53ml, p = 0.19) changes in all the patients and subgroup analyses of MCC in patients with either interstitial cystitis (IC) or detrusor overactivity (DO) (C; MCC mean difference for IC −19.10 ml, 95% CI −59.37 to 21.17 ml, p = 0.35; MCC mean difference for DO 53.36 ml, 95%CI 15.42 to 91.29 ml, p = 0.006).

Compared to the control, RTX treatment could not increase the FDC in the patients. No statistical difference existed between the two groups (mean difference −16.56 ml, 95% CI −55.79 to 22.67 ml, p = 0.41, Figure. 5A). Our data showed that RTX treatment increased MCC by 34 ml (95% CI −16.54 to 84.53 ml, p = 0.19, Figure. 5B) in the total LUTS patients. When the response to RTX treatment in patients with different causes of LUTS was further analyzed, the results indicated that RTX treatment could significant increase MCC in patients with DO (mean MCC increase 53.36 ml, 95%CI 15.42 to 91.29 ml, p = 0.006, Figure. 5C). However, no significant MCC increase was noted in patients with IC (mean MCC increase −19.10 ml, 95% CI −59.37 to 21.17 ml, p = 0.35; Figure. 5C).

### 4. Adverse outcomes

The main adverse outcome of RTX treatment was a warm sensation of the bladder during instillation. This discomfort was mild and well tolerated, and usually lasted for one hour. No significant changes in results of hematology or biochemistry analyses were noted after RTX treatment. No other serious adverse events were reported in all the included trails.

### 5. Sensitivity analyses

**Figure 6:**
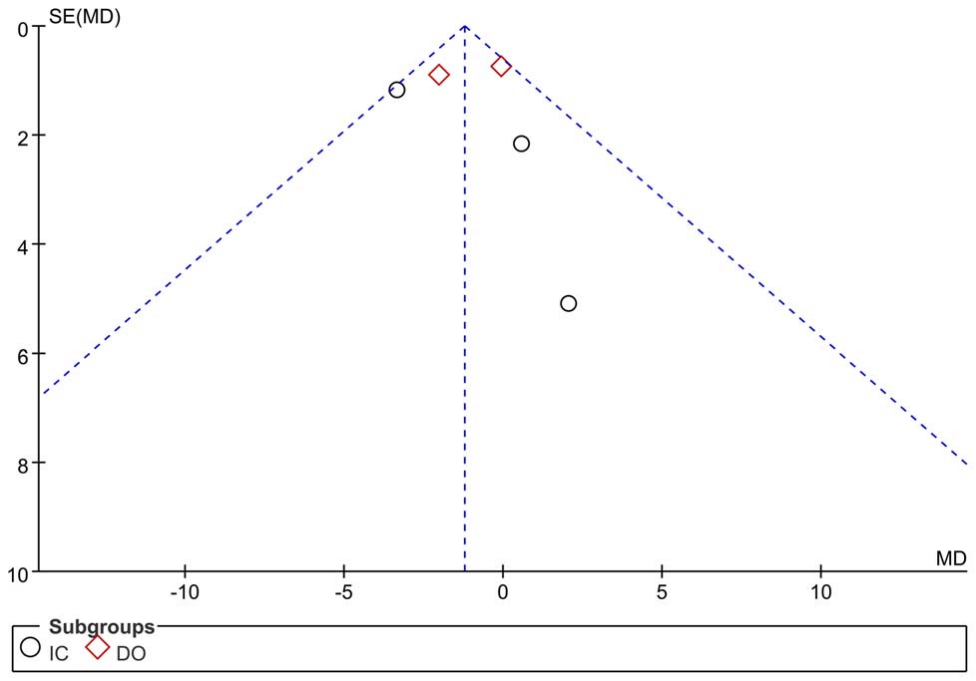
Funnel plot of studies indacated that there was no significant publication bias in this meta-analysis. The mean difference (MD) of urinary frequency and its standard error (SE) for Resin iferatoxin effect estimate in patients with interstitial cystitis (IC) and detrusor overactivity (DO) were plotted on the horizontal axis and vertical axis respectively. A symmetrical plot suggests no publication bias exist in the meta-analysis.

The funnel plots revealed that no significant publication bias existed in the present meta-analysis (Figure. 6). Heterogeneity calculation indicated that the heterogeneity didn't exist in analyses of bladder pain (I^2^ = 21%), frequency (I^2^ = 1%), nocturia (I^2^ = 0%) or FDC (I^2^ = 0%). However, high heterogeneity existed in the analyses of incontinence (I^2^ = 87%) and MCC (I^2^ = 65%). Subgroup analysis of MCC showed that heterogeneity didn't exist in the DO group (I^2^ = 5%).

### 6. Quality assessment

**Figure 7:**
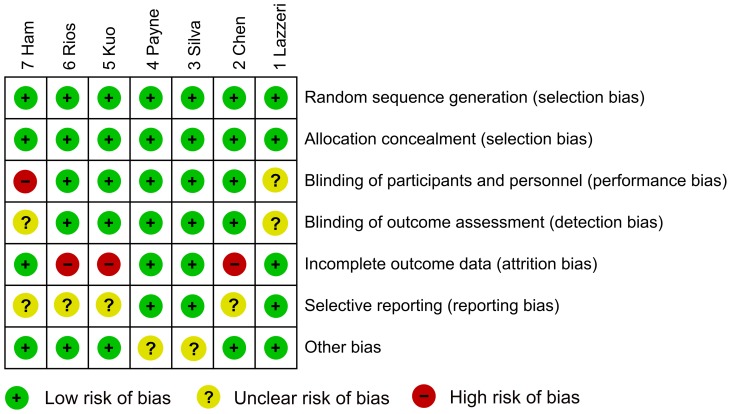
Quality assessment in this meta-analysis demonstrated a low risk of bias in five studies (Rios 2007, Kuo 2006, Payne 2005, Silva 2005 and Chen 2005) and a relatively high risk of bias in the remaining two studies (Ham 2012 and Lazzeri 2000).

The methodological quality of the seven included studies is illustrated in Figure 7. The results indicated that the five randomized, double-blind, placebo controlled studies had a low risk of bias [20]–[24]; whereas the remaining two prospective, randomized, placebo controlled studies had a relatively high risk of bias [19], [27].

## Discussion

Symptoms of bladder pain, frequency, nocturia, urgent incontinence and bladder dysfunction are highly prevalent among LUTS patients with either IC or DO. Although treatments have been developed for a long time, urologists still often experience refractory and treatment-resistant patients on whom current therapies have very limited effects [2], [3], [36]. In recent years, several novel approaches with potential benefit for patients, including psychosocial, behavioral, physical and oral treatments, intravesical treatment, electric neuromodulation, reconstructive surgery, and gene therapy, have been clinically used [3], [15], [36]. Systematic review should be performed to fully evaluate the effect of each of these approaches and to provide important evidence for best-practice decisions in these patients. Since intravesical desensitization of TRPV1 with RTX has been considered as a treatment with great potential for patients with storage LUTS [13], [18], we performed this meta-analysis to combine the results from multiple studies and further evaluate the efficacy of intravesical RTX, on bladder pain, urinary frequency, nocturia, incontinence and urodynamic parameters (FDC and MCC), when used for the treatment of storage LUTS in patients with either IC or DO.

### 1. RTX on bladder pain

Our analysis indicated that bladder pain in patients with either IC or DO was significantly reduced after intravesical RTX treatment. One mechanism might be the desensitization of bladder afferent C fibers after RTX binds to TRPV1. Recent data suggest that TRPV1 is mainly expressed in C fibers and regulates the frequency of bladder reflex contractions [11]. Activation of type C bladder afferent fibers could lead to the enhancements of local bladder segmental contractility and the spinal micturition reflex, and therefore result in the development of storage LUTS [3], [4], [7]–[9]. RTX, as a potent agonist of TRPV1, can bind to TRPV1, inactivate bladder C fibers, prevent the input of bladder sensory signals, inhibit local bladder segmental contractility and the spinal micturition reflex, and finally improve bladder pain [9], [13], [37].

Another mechanism of RTX in the relief of bladder pain might be the inhibition of neuropeptides, including CGRP and SP, which play an important role in generation of peripheral neurogenic inflammation and maintenance of bladder pain in IC and DO patients. It was reported that CGRP and SP are expressed in a majority of TRPV1 fibers in the rat bladder [38]. Alterations in SP-immunoreactivity and SP receptor mRNA have been observed in bladder biopsies from patients with IC [39], [40]. Release of neuropeptides from afferent terminals could lead to the occurrence of neurogenic inflammation and increase C fibers afferent sensitivity at the peripheral sites in these patients [41]. Intravesical administration of RTX could temporarily decrease (for approximately four weeks) the number of SP- and CGRP-immunoreactive fibers in the rats [42]. In addition, intravesical RTX might result in a remarkable downregulation of TRPV1 expression in bladder C fibers and decrease the sensitivity of bladder pain [15]. The long-term (more than 3 months) effects of RTX on inhibition of neuropeptides and TRPV1 expression in afferent C fibers and subsequent bladder pain need to be further investigated.

The outcome of intravesical RTX on bladder pain in storage LUTS patients was controversial. In clinical trials, Ham et al reported that intravesical RTX instillation could significantly improve IC-related bladder pain after 3 months of treatment [19]. In contrast, Chen et al reported that no significant improvement of IC-related pain was observed in 22 patients with IC at a 12-week follow-up after RTX treatment [24]. Although the results from multiple studies in our meta-analysis demonstrated a statistically significant decrease (p<0.05) of bladder pain in IC and DO patients, the absolute value of VAS decrease was very small with less clinical benefit (<1.0 in VAS decrease). One possible explanation is the complexity of the primary bladder afferent fibers. In general, bladder C fibers mainly conduct chemical and thermal stimuli that in turn cause nociceptive responses. TRPV1-expressing sensory C fibers might not be involved in the development of mechanical allodynia. A relatively common symptom of neuropathic pain is tactile allodynia which can be mediated by Aβ fibers. For this reason, intravesical RTX that targets C fibers might not be able to block the input of mechanical allodynia due to the hyperactivation of Aβ fibers, and innocuous mechanical stimuli could be perceived as painful in patients with IC or DO [43].

### 2. RTX on urinary frequency, nocturia and incontinence

Historical literature also indicated that the clinical effect of intravesical RTX on the outcomes of urinary frequency, nocturia and incontinence in patients with storage LUTS was controversial. Some investigations reported significant decreases of urinary frequency, nocturia and incontinence [16], [22], [26], [27], [29], [31]; while others demonstrated no significant improvement in LUTS in these patients [19], [20], [23], [27]. In this study, we included seven eligible studies and the results of meta-analysis showed that no significant improvement in urinary frequency, nocturia or incontinence was noted after RTX therapy [19]–[24], [27] (Table 1).

The mechanisms underlying altered TRPV1 expression and sensitivity are likely to be very complex, involving additional factors that may be altered in human bladder urothelial cells. Both human and animal studies had shown that altered production of urothelial-derived factors, such as nerve growth factor (NGF), adenosine triphosphate and acetylcholine, could influence afferent excitability as well as activity of bladder smooth muscle [44]. Elevated urinary levels of NGF had been found in patients with idiopathic and neurogenic DO [45]. Further studies showed that NGF could enhance the expression and sensitivity of TRPV1, supporting the NGF-dependent roles in sensitization of mechanosensitive bladder afferents and development of detrusor overactivity [44], [46]. Hence, it might be reasonable to combine an approach to prevent NGF-dependent bladder sensory excitability and spinal micturition reflex [15].

P2X3 receptor also plays an important role in C-fiber afferent pathways. adenosine triphosphate released from bladder urothelium is able to affect its P2X3 receptor and play a prominent role in the primary afferent sensitization and development of LUTS [47]. More recently, preclinical studies found that the use of small molecule compounds targeting the P2X3 receptor have the potential of providing relief for patients suffering storage, voiding and sensory symptoms [48]. In addition, muscarinic receptors (i.e. M2 and M3 receptors) are thought to play a key role in urinary bladder contraction. The disease condition with IC or DO might alter the expression of muscarinic receptors in the bladder, which could subsequently impact the release of transmitters, such as acetylcholine, from the bladder urothelial and suburothelial tissue and eventually result in alterations in bladder contractility as well as afferent sensation [44].Therefore, further study of muscarinic receptor subtypes and associated intracellular signaling mechanisms would be beneficial for the development of therapies for controlling storage LUTS in patients with IC or DO [15], [44].

Taken together, the pathogenesis of storage LUTS might be extremely complex and multifactorial. Blockade of TRPV1 in the bladder lumen using intravesical RTX might not be able to completely inhibit the sensory input of bladder, enhancement of local segmental contractility, or development of storage LUTS. This may be the reason why monotherapy with intravesical RTX cannot improve urinary frequency, nocturia and incontinence in the patients with either IC or DO. In the future, a better understanding of the multiple mechanisms of storage LUTS and use of a combination of inhibitors in different pathways might be able to provide an optimum paradigm to manage storage LUTS in these patients.

### 3. RTX on urodynamic parameters

The clinical effect of RTX on bladder capacity might be selective in patients with storage LUTS. Kuo et al reported that the bladder capacity was significantly increased three months after RTX treatment in a total of 54 patients with DO refractory to anticholinergics [21]. Silva et al also reported that intravesical RTX was effective in increasing bladder capacity in patients with DO of spinal origin [22]. However, RTX was not effective in patients with IC [23]. In our meta-analysis, the results further confirmed that MCC was significantly improved in patients with DO after RTX treatment, while no significant improvement of MCC was noted in patients with IC. The selective effect of RTX on bladder capacity observed between patients with IC and DO imply that TRPV1 might play a more important role in the development of the disease in the DO patients than in the IC patients. It was reported that bladder TRPV1 immunoreactivity was significantly increased in patients with DO, and was decreased significantly after intravesical RTX treatment [11]. Our data also indicated that FDC was not significantly improved in patients with storage LUTS after RTX therapy. One reason might be that RTX could not change the bladder reflex sensitivity when the bladder is filling [13].

### 4. Statistical heterogeneity

Statistical heterogeneity didn't exist in the bladder pain, frequency, nocturia and FDC analyses. However, high heterogeneity existed in the incontinence and MCC analyses. We tried to find the reason for the heterogeneity by removing one of the trails. In incontinence, the heterogeneity did not remarkably changed by removing one trial from three included trails. In the “Kuo 2006” trail, incontinence was recorded by episodes/three-day voiding diary and others by a one-day voiding diary [19]. This may be one of the reasons for the heterogeneity. However, the heterogeneity disappeared in subgroup analysis of MCC, implying that the kind of diseases might affect the heterogeneity for subanalysis.

### 5. Limitations of this meta-analysis

To the best of our knowledge, this research represents the first systematic review comparing RTX with controls in the treatment of storage LUTS. The data from all studies that met our predefined criteria was included in this analysis, and the results are helpful for clinical practices. However, we also acknowledge some inherent limitations in this meta-analysis that cannot be ignored when interpreting our data. Firstly, most studies included in this analysis were small scale studies. Secondly, there were differences in the length of the follow-up period for patients, ranging from 4 to 12 weeks after therapy. Furthermore, different follow-up schemes were detected among the included studies, so that the use of a standard follow-up scheme and results from long-term (more than 3 months) follow-up studies need to be further investigated. Thirdly, the dosage of RTX used in the reviewed studies was not identical, which might lead to different outcomes in LUTS patients. Fourthly, the heterogeneity existed in urinary incontinence and MCC. Subgroup analyses by different causes of LUTS could not be achieved because of insufficient data. Fifthly, RTX is an unstable chemical. It is susceptible to slow degradation if exposed to air. It will develop a few tenths of a percent of impurities over a period of several months during storage if air is not rigorously excluded from its container. RTX has also been demonstrated to be able to absorb to polyethylene, polyvinylchloride, and latex (but not silicone or glass) catheters and containers. Furthermore, the activity of RTX in solution will be lost within a few hours if RTX is stored in a plastic container. Thus, the clinical benefits might be limited due to the uncertainty of the quality of the RTX before instillation. Differences in outcomes of intravesical RTX in different studies might partly result from different ways of preparing and storing RTX [49]. Due to these limitations mentioned above, our findings of the clinical effect of RTX on storage LUTS patients in this study should be interpreted with caution.

## Conclusions

This meta-analysis indicated that RTX could significantly improve bladder pain in LUTS patients with either IC or DO, and increase MCC in patients with DO. No significant improvement was observed in frequency, nocturia, incontinence or FDC. These findings implied that intravesical RTX treatment used in combination with currently available therapeutic approaches might be beneficial for some selected patients with either IC or DO. Given the limitations in the small trial size and risk of bias in the included studies, a large, multicenter, well-designed random control trial with a long-term follow-up is needed to further confirm our findings. At present, great caution should be taken when intravesical RTX is used in patients with either IC or DO.

## Supporting Information

Checklist S1 **PRISMA checklist.**
(PDF)
