# Supplementary material for: Intravesical Resiniferatoxin for the Treatment of Storage Lower Urinary Tract Symptoms in Patients with Either Interstitial Cystitis or Detrusor Overactivity: A Meta-Analysis
Source: PLoS One. 2013 Dec 20;8(12):e82591. doi: 10.1371/journal.pone.0082591 (PMC3869704; doi:10.1371/journal.pone.0082591)
Supplement: Checklist S1 — PRISMA checklist. (PDF) [file pone.0082591.s001.pdf]

PRISMA 2009 Checklist

Section/topic	#	Checklist item	Reported on page #
**TITLE**			
Title	1	Identify the report as a systematic review, meta-analysis, or both.	Title
**ABSTRACT**			
Structured summary	2	Provide a structured summary including, as applicable: background; objectives; data sources; study eligibility criteria, participants, and interventions; study appraisal and synthesis methods; results; limitations; conclusions and implications of key findings; systematic review registration number.	Abstract
**INTRODUCTION**			
Rationale	3	Describe the rationale for the review in the context of what is already known.	Introduction
Objectives	4	Provide an explicit statement of questions being addressed with reference to participants, interventions, comparisons, outcomes, and study design (PICOS).	Introduction
**METHODS**			
Protocol and registration	5	Indicate if a review protocol exists, if and where it can be accessed (e.g., Web address), and, if available, provide registration information including registration number.	Search Strategy
Eligibility criteria	6	Specify study characteristics (e.g., PICOS, length of follow-up) and report characteristics (e.g., years considered, language, publication status) used as criteria for eligibility, giving rationale.	Search Strategy, Participants and Interventions
Information sources	7	Describe all information sources (e.g., databases with dates of coverage, contact with study authors to identify additional studies) in the search and date last searched.	Search Strategy
Search	8	Present full electronic search strategy for at least one database, including any limits used, such that it could be repeated.	Search Strategy
Study selection	9	State the process for selecting studies (i.e., screening, eligibility, included in systematic review, and, if applicable, included in the meta-analysis).	Search Strategy, Participants and Interventions
Data collection process	10	Describe method of data extraction from reports (e.g., piloted forms, independently, in duplicate) and any processes for obtaining and confirming data from investigators.	Search Strategy, Participants and Interventions, Data Extraction
Data items	11	List and define all variables for which data were sought (e.g., PICOS, funding sources) and any assumptions and simplifications made.	Data Extraction,<br>Quality Assessment
Risk of bias in individual studies	12	Describe methods used for assessing risk of bias of individual studies (including specification of whether this was done at the study or outcome level), and how this information is to be used in any data synthesis.	Data Extraction,<br>Quality Assessment
Summary measures	13	State the principal summary measures (e.g., risk ratio, difference in means).	Data Extraction
Synthesis of results	14	Describe the methods of handling data and combining results of studies, if done, including measures of consistency (e.g., I^2) for each meta-analysis.	Data Extraction, Quality Assessment

PRISMA 2009 Checklist

Section/topic	#	Checklist item	Reported on page #
Risk of bias across studies	15	Specify any assessment of risk of bias that may affect the cumulative evidence (e.g., publication bias, selective reporting within studies).	Data Extraction, Quality Assessment
Additional analyses	16	Describe methods of additional analyses (e.g., sensitivity or subgroup analyses, meta-regression), if done, indicating which were pre-specified.	Data Extraction, Quality Assessment
**RESULTS**			
Study selection	17	Give numbers of studies screened, assessed for eligibility, and included in the review, with reasons for exclusions at each stage, ideally with a flow diagram.	Search Strategy, Participants and Interventions, Figure 1
Study characteristics	18	For each study, present characteristics for which data were extracted (e.g., study size, PICOS, follow-up period) and provide the citations.	Participants and Interventions, Data Extraction,Table 1
Risk of bias within studies	19	Present data on risk of bias of each study and, if available, any outcome level assessment (see item 12).	Sensitivity analyses, Quality assessment
Results of individual studies	20	For all outcomes considered (benefits or harms), present, for each study: (a) simple summary data for each intervention group (b) effect estimates and confidence intervals, ideally with a forest plot.	Sensitivity analyses, Table 1
Synthesis of results	21	Present results of each meta-analysis done, including confidence intervals and measures of consistency.	Outcomes of bladder pain; Outcomes of urinary frequency, nocturia and incontinence; Outcomes of urodynamic parameters
Risk of bias across studies	22	Present results of any assessment of risk of bias across studies (see Item 15).	Sensitivity analyses, Quality assessment
Additional analysis	23	Give results of additional analyses, if done (e.g., sensitivity or subgroup analyses, meta-regression [see Item 16]).	Adverse outcomes, Sensitivity analyses, Quality assessment
**DISCUSSION**			
Summary of evidence	24	Summarize the main findings including the strength of evidence for each main outcome; consider their relevance to key groups (e.g., healthcare providers, users, and policy makers).	RTX on bladder pain; RTX on urinary frequency, nocturia and incontinence; RTX on urodynamic parameters
Limitations	25	Discuss limitations at study and outcome level (e.g., risk of bias), and at review-level (e.g., incomplete retrieval of identified research, reporting bias).	Limitations of this meta-analysis
Conclusions	26	Provide a general interpretation of the results in the context of other evidence, and implications for future research.	Conclusions
**FUNDING**			
Funding	27	Describe sources of funding for the systematic review and other support (e.g., supply of data); role of funders for the systematic review.	Funding

From: Moher D, Liberati A, Tetzlaff J, Altman DG, The PRISMA Group (2009). Preferred Reporting Items for Systematic Reviews and Meta-Analyses: The PRISMA Statement. PLoS Med 6(6): e1000097. doi:10.1371/journal.pmed1000097 For more information, visit: **www.prisma-statement.org**.
